# Synthesis of Me Doped Mg(OH)_2_ Materials for Thermochemical Heat Storage

**DOI:** 10.3390/nano8080573

**Published:** 2018-07-26

**Authors:** Elpida Piperopoulos, Marianna Fazio, Emanuela Mastronardo

**Affiliations:** 1 Department of Engineering, University of Messina, 98166 Messina, Italy; faziom@unime.it; 2 National Interuniversity Consortium of Materials Science and Technology (INSTM), 50121 Firenze, Italy; 3 Institute of Advanced Studies of Madrid (IMDEA), Thermochemical Processes Unit, 28935 Madrid, Spain; emanuela.mastronardo@northwestern.edu; 4 Department of Materials Science and Engineering, Northwestern University, Evanston, IL 60208, USA

**Keywords:** magnesium hydroxide, thermochemical heat storage, metal doping

## Abstract

In order to investigate the influence of metal (Me) doping in Mg(OH)_2_ synthesis on its thermochemical behavior, Ca^2+^, Co^2+^ and Ni^2+^ ions were inserted in Mg(OH)_2_ matrix and the resulting materials were investigated for structural, morphological and thermochemical characterization. The densification of the material accompanied by the loss in porosity significantly influenced the hydration process, diminishing the conversion percentage and the kinetics. On the other hand, it increased the volumetric stored/released heat capacity (between 400 and 725 MJ/m^3^), reaching almost three times the un-doped Mg(OH)_2_ value.

## 1. Introduction

The Renewable Energy Directive establishes an overall policy for the production and promotion of energy from renewable sources in the European Union (EU). The EU target for 2020 is to achieve at least 20% of its total energy requests with renewables. EU countries have already agreed on a new renewable energy target of at least 27% as climate goals for 2030. On 30 November 2016, the European Commission published a proposal for a revised Renewable Energy Directive to make the EU a global leader in renewable energy. Renewable energy can be produced from a wide variety of sources including solar, wind, hydro, geothermal, tidal and biomass. By using more renewables to meet its energy needs, the EU lowers its dependence on imported fossil fuels and makes its energy production more sustainable. Due to climatic variability, the means of storing these types of renewable energy have become an urgent consideration [1]. This has led to the search for efficient and sustainable methods of storing energy and a considerable effort to understand how energy storage works, how existing methods can be improved and how new ones can be developed. Thermal energy storage (TES) transfers heat to storage media during the charging period and releases it at a later stage during the discharging step. It can be usefully applied in solar plants, or in industrial processes. Through TES systems, heat can be stored in the form of sensible [2] or latent heat [3] or in the form of chemical energy (thermochemical storage) [4]. Sensible heat storage is achieved by varying the temperature of a storage material. Latent heat storage is realized changing a material phase at a constant temperature, while the thermochemical storage promotes a reversible chemical reaction. Sensible heat storage is well-documented. Latent heat storage, using phase change materials (PCMs), has been heavily researched and is widely used domestically and industrially. Thermochemical heat storage (TCS) is still at an early stage of laboratory and pilot research in spite of its attractive application for long-term energy storage and higher stored/released heat values [5,6]. Storage density, in terms of the amount of energy per unit of volume, is important for optimizing the use of these kind of materials [7] as it is relevant to their transportation and application in concentrated systems [6]. In 1978, Bowery et al. [8] investigated the practical feasibility of a BaO_2_/BaO system for high-temperature heat storage. Theoretical calculations discovered that the endothermic reaction occurred when the temperature exceeds 754 °C, and the calculated energy storage density was about 2.9 GJ/m^3^. Subsequently, the reaction was found difficult to achieve complete conversion; even if the temperature rose to 1027 °C, the theoretical conversion rate of BaO_2_ had a maximum of 85%. Since then, several TCS materials have been studied and many strategies have been adopted to improve these storage materials [9]. Carrillo et al. studied the effect that co-doping of Mn oxides with Fe and Cu has on the redox temperatures of both forward and reverse reactions [10,11]. Block et al. tested several compositions of eight binary metal oxide systems as well as the pure metal oxides (cobalt oxide, iron oxide, copper oxide and manganese oxide) in terms of their ability to store energy thermochemically [12]. The calcium oxide hydration/dehydration reaction is proposed as a suitable reaction couple for thermochemical energy storage systems for its high energy density (0.4 kWh/kg) and low material cost (50 €/t) [13,14,15]. Sakellariou et al. prepared mixed calcium oxide–alumina compositions, assessed in terms of their cyclic hydration–dehydration performance in the temperature range of 200–550 °C. One of the main purposes of using Al as additive was related to materials structural enhancement [16]. A suitable TCS system storing in lower temperature range between 200 °C and 400 °C, which has been examined in this study, is the dehydration/hydration reaction of magnesium hydroxide/oxide:

Mg(OH)_2_(s) ↔ MgO(s) + H_2_O(v) ΔH_0_ = ±81 kJ/mol (1)

The above system offers several advantages, high storage capacity, medium operating temperature range (as reported above), long-term storage of reactants and products, low heat loss and non-toxicity of the materials [17]. Through the endothermic dehydration reaction, heat can be stored and released when required by the reverse exothermic hydration reaction. This system has been widely studied to improve storage material performances, as mass and volume energy density, kinetics and ciclability. Shkatulov et al. studied LiNO_3_-doped Mg(OH)_2_ storage material that exhibits a decrease in the dehydration start temperature by 76 °C [18]. Junichi et al. developed a 6.8 wt.% LiCl/Mg(OH)_2_ system that drops the dehydration temperature of magnesium hydroxide, from 277 °C to 233 °C, being able to store 816 MJ/m^3^ volumetric heat storage capacity [19]. Muller et al. found that calcium doping of magnesium oxide results in significantly increased water dissociation rates, thus enhancing both hydration rate and reaction completeness of hydration compared to pure MgO [20]. Zamengo et al. prepared a Mg(OH)_2_/MgO system supported on expanded graphite. The pelletized storage material, decreasing the tablets volume required to store the same amount of thermal energy of Mg(OH)_2_ pellets of almost 13.6%, increases volume energy density [21]. In previous studies it was found that, synthesizing Mg(OH)_2_ in presence of a cationic surfactant (cetyl trimethyl ammonium bromide—CTAB), an optimum CTAB concentration exists and it exhibits the highest volumetric stored/released heat capacity, ∼560 MJ/m^3^ two times higher than that measured over Mg(OH)_2_ prepared in absence of CTAB [22]. The purpose of this work is to investigate the influence of metal (Ca^2+^, Co^2+^ and Ni^2+^) doping in Mg(OH)_2_ synthesis on its structural and morphological properties and consequently on its thermochemical behavior.

## 2. Materials and Methods 

### 2.1. Samples Preparation

The Metal (Me) doped Mg(OH)_2_ samples were synthesized by precipitation method. The following raw materials were used: Mg(NO_3_)_2_∙6H_2_O, 99%, supplied by Sigma-Aldrich (St. Louis, MO, USA), as magnesium source, ammonia solution (NH_4_OH, 30 wt.% Carlo Erba) as precipitating agent and Ca(NO_3_)_2_, Co(NO_3_)_2_ and Ni(NO_3_)_2_ respectively for Ca^2+^, Co^2+^ and Ni^2+^ doping metals. The precipitation was carried out as follows: 50 mL of a solution containing Mg^2+^ and Me ion (Ca^2+^ or Co^2+^ or Ni^2+^) were gradually added (2.5 mL/min) through a peristaltic pump to 150 mL of NH_4_OH solution (*ph* = 11.8), under magnetic stirring. The final solution was aged at ambient temperature for 24 h, then it was vacuum filtered (0.22 μm); the collected solid was washed with deionized water and dried in a vacuum oven (Binder, Tuttlingen, Germany) at 50 °C overnight. Table 1 reports the code of samples and the chemical composition of solutions for all the preparations.

### 2.2. Samples Characterization

Quantitative analysis of calcium, nickel and cobalt present into the solid was performed by means of ICP-MS spectrometer (PERKIN-ELMER, model NexION 300×, Waltham, MA, US). Approximately 3 mg (*wt_measured_*) of each synthesized sample (*wt_synthesized_*) was dissolved in the minimum volume of concentrated HNO_3_, and then deionized water was added until the final volume of 10 mL (*V_f_*) was reached. Exactly 100 μL (*V*_1_) of this solution, mixed of 100 μL of concentrated HNO_3_, were diluted up to 10 mL (*V*_2_) and then analyzed. The grams of dopant (*Me*) present in the samples are calculated as follows:

(2) $$Me\;\left(g\right)=\left\{\left(\frac{{\left[Me\right]}_{ICPMS}\;\left(\mathrm{ppm}\right)\times V_{2}\left(l\right)}{V_{1}\;\left(l\right)}\right)\times \frac{V_{f}\left(l\right)}{1000}\right\}\times \frac{{wt}_{synthesized}\;\left(g\right)}{{wt}_{measured}\;\left(g\right)}$$

Pore volume was calculated by Barrett-Joyner-Halenda (BJH) method using the nitrogen desorption isotherm measured at −196 °C with a Quantachrome Autosorb-iQ MP (NOVA 1200, Boynton Beach, FL, USA) instrument. Samples were degassed prior to analysis under vacuum at 120 °C for 3 h. Each sample’s mean particle size was determined by Dynamic Light Scattering (DLS) technique. DLS was measured at 25 °C using a Zetasizer Nano ZS instrument (Malvern Instruments, Malvern, UK) equipped with a helium-neon 4 mW laser (wavelength *λ*_0_ = 632.8 nm). The scattering angle was equal to 173°. Prior to measurements, samples were sonicated for 30 minutes in ethylene glycol. The bulk density of samples was measured by weighing a known volume of solids (*V* (mL)) and calculated by the formula:

(3) $${\rho =m\;(\mathrm{kg})/V(m}^{3})$$

The as-prepared samples were analyzed by means of scanning electron microscopy (SEM, Quanta 450, FEI, Hillsboro, OR, USA) and X-Ray Diffraction (XRD, Bruker D8 Advance, Bruker, Billerica, MA, USA) to determine their morphology and crystal structure.

SEM analysis were performed on Cr-metallized samples and operating with an accelerating voltage of 10 kV under high vacuum conditions (6.92 × 10^−5^ Pa).

### 2.3. Thermochemical Performance

The evaluation of the thermochemical behavior of the prepared samples under cyclic heat storage/release experiments was performed using a customized thermogravimetric unit (STA 449 F3 Jupiter Netzsch, Selb, Bavaria, Germany) that allowed us to carry out a succession of dehydration and hydration reactions. The thermogravimetric apparatus was equipped with a water vapor generator for the vapor supply during the hydration reaction. A cyclic heat storage/release experiment was carried out on a mass of ~15 mg as reported elsewhere [17,23,24]: the sample was first dried at 125 °C in inert atmosphere (under N_2_ flow: 100 mL/min) for 60 min to remove the physically adsorbed water. Then, the temperature was increased at 10 °C/min up to the dehydration temperature (*T*_d_ = 350 °C) and dehydration reaction proceeded over 120 min under isothermal conditions. After the complete dehydration reaction, the temperature was decreased (cooling rate = −10 °C/min) to the hydration temperature (*T*_h_ = 125 °C). The hydration reaction proceeded over 120 min, during which the water vapor necessary for the re-hydration reaction was supplied by the water vapor generator at 2.2 g/h and mixed with 35 mL/min N_2_ as carrier gas (*p*_H2O_ = 57.8 kPa). After the fixed hydration time, the water vapor supply was stopped and the sample was kept at 125 °C for 30 min under a constant N_2_ flow (100 mL/min) to remove physically adsorbed water from the sample. This procedure was repeated for each heat storage/release cycle. In this study, for a preliminary comparison, the samples were subjected to 3 cycles experiments. To be consistent with previous studies [17,22,23,25] the materials performances were expressed in terms of reacted fraction (*β*(%)) defined by Equation (4):

(4) $$\beta (\%)=(1-\frac{\Delta m_{\mathrm{real}}}{\Delta m_{\mathrm{th}}})\times 100,$$

where Δ*m_real_*(%) was the instantaneous real mass change and Δ*m_th_*(%) was the theoretical mass change due to the dehydration of 1 mol Mg(OH)_2_, respectively expressed by Equations (5) and (6):

(5) $$\Delta m_{\mathrm{real}}\left(\%\right)=\frac{m_{\mathrm{in}}-m_{\mathrm{inst}}}{m_{\mathrm{in}}}\times 100,$$

(6) $$\Delta m_{\mathrm{th}}\left(\%\right)=\left(\frac{M_{\mathrm{Mg}\left(\mathrm{OH}\right)2}-M_{\mathrm{MgO}}}{M_{\mathrm{Mg}\left(\mathrm{OH}\right)2}}\times 100\right)=30.89\%,$$

where *m_in_*(*g*) and *m_inst_*(*g*) were respectively the initial sample mass and the instantaneous mass during TG analysis. While, *M_Mg_*_(*OH*)2_(g/mol) and *M_MgO_*(g/mol) were respectively the molecular weight of Mg(OH)_2_ and MgO.

The dehydration and hydration conversions (∆*β*_*d*/*h*_(%)) were calculated respectively by Equations (7) and (8):

(7) $$\Delta \beta_{d}\left(\%\right)=\beta_{d}^{i}-\beta_{d}^{f},$$

(8) $$\Delta \beta_{h}\left(\%\right)=\beta_{h}-\beta_{d}^{f},$$

where $\beta_{d}^{i}$ and $\beta_{d}^{f}$ were respectively the reacted fraction at the beginning and at the end of the dehydration treatment. While, *β_h_* was the final reacted fraction of MgO at the point of water supply termination.

The stored/released heat capacity per volume unit ($Q_{s/r}^{V}$ (MJ/m^3^)) was calculated using Equation (9):

(9) $$Q_{s/r}^{V}\;\left({\mathrm{MJ}/m}^{3}\right)=-\frac{\Delta H^{0}}{M_{\mathrm{Mg}\left(\mathrm{OH}\right)2}}\times \Delta \beta_{d/h}\times \rho ,$$

where Δ*H*^0^ (kJ/mol) is the enthalpy of reaction and *ρ* (kg/m^3^) the bulk density of the sample.

## 3. Results and Discussion

### 3.1. Me Doped Mg(OH)_2_ Preparation

In the first instance, it was evaluated whether, under the preparation condition of the present work, each ion could precipitate as hydroxide. Precipitation of hydroxide from the solution through the reaction (10)

Me*^n^*^+^ + *n*OH^−^ → Me(OH)*_n_* (s), (10)

occurs when the supersaturation conditions are reached. Supersaturation conditions are defined as:

[Me*^n^*^+^]·[OH^−^]*^n^* > K_sp_, (11)

where [Me*^n^*^+^] and [OH^−^] represent the concentration expressed as molarity (M) of cation and hydroxyl ions, *n* represent the hydroxyl’s stoichiometric coefficient and K_sp_ is the thermodynamic equilibrium constant of solubility product. As shown in Table 2 supersaturation conditions are satisfied in case of Mg(OH)_2_, Co(OH)_2_ and Ni(OH)_2_ formation but not for Ca(OH)_2_, whatever the calcium concentration used being the ionic product [Me*^n^*^+^]·[OH^−^]*^n^* < K_sp_.

**Figure 1:**
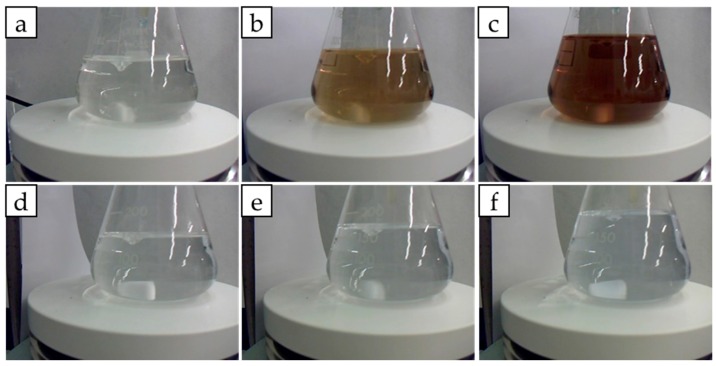
Formation of cobalt and nickel hexamine complexes. Starting aqueous Co^2+^ solution 0.002 M (**a**); Upon addition of NH_4_(OH) (**b**) and after mixing for 24 h (**c**). Starting aqueous Ni^2+^ solution 0.002 M (**d**); Upon addition of NH_4_(OH) (**e**) and after mixing for 24 h (**f**).

As will be further explained, in reality neither Ni(OH)_2_ nor Co(OH)_2_ solids form (Figure 1). This is due to the fact that with a large excess of ammonia, cobalt and nickel hydroxides redissolve forming hexaminocobalt(II) (Co(NH_3_)_6_^2+^) and hexaminonickel(II) (Ni(NH_3_)_6_^2+^) ions as ammonia substitutes as a ligand [25]. As shown in Figure 1 no solid formation occurs even after 24 h. In case of Co^2+^ solution, pink colored due to presence of Co(H_2_O)_6_^2+^, upon addition of NH_4_OH color rapidly changes to yellow then to a deep red-brown. This is due to oxidation of hexaminocobalt(II) to hexaminocobalt(III) ions by air [25]. In case of Ni^2+^, light green colored by the complex Ni(H_2_O)_6_^2+^, addition of ammonia causes a color change to light blue typical of Ni(NH_3_)_6_^2+^ complex [25].

**Figure 2:**
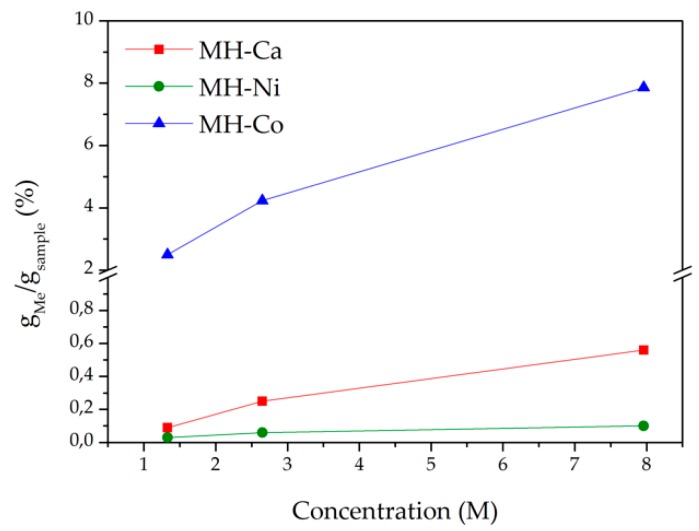
Me content per g of sample obtained by precipitation vs. Me concentration in the starting solution.

After these preliminary evaluations MH, MH-Ca, MH-Co, and MH-Ni were prepared according to the procedure reported in the experimental section. The Me content on the final sample, (*g_Me_*/*g_sample_*)%, is reported in Figure 2. As general feature, regardless the type of Me, the load increases with the initial amount present into the solution. At given Me initial concentration the *g_Me_*/*g_sample_* content varies among the different type of Me; in case of Ni the lowest amount of loaded Me is obtained while the highest amount is achieved for Co containing samples. 

Considering that no calcium, cobalt and nickel hydroxide form, it can be assumed that these metal ions are included into Mg(OH)_2_ host matrix.

### 3.2. Structure and Morphology of Samples

**Figure 3:**
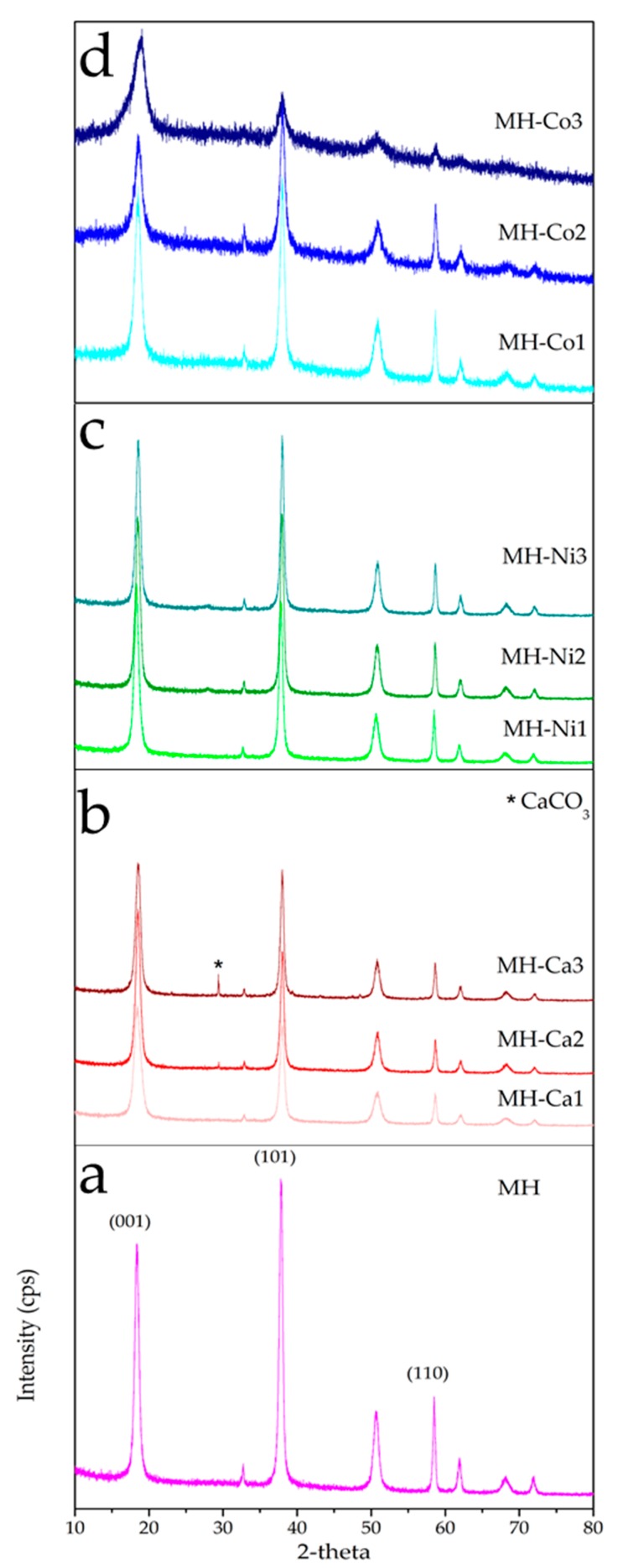
XRD patterns of MH (**a**), MH-Ca (**b**), MH-Ni (**c**) and MH-Co (**d**) samples.

In Figure 3a–d XRD analysis of MH and MH-Me samples are shown. The diffractograms are acquired in a 2θ range between 10° and 80°. Mg(OH)_2_ spectrum (Figure 3a) presents the reflection peaks typical of hexagonal brucite (2θ: 18.5°, 32.5°, 38°, 51°, 58.5°, 62°, 68°, 72°), in agreement with standard data, (JCPDS 7-0239 and JCPDS 25-0284). The three most intense peaks (2θ = 18.5°, 38.0°, 58.5°) appear sharp and narrow as a result of high degree of crystallization of hexagonal brucite. Reflection peaks of MH-Ca and MH-Ni samples, regardless the amount of calcium or nickel, match with those of pure Mg(OH)_2_ in terms of peaks position (Figure 3b,c). They are intense and narrow thus suggesting that the high crystallization degree of brucite is maintained. For MH-Ca2 and MH-Ca3 samples (Figure 3b) is also present a peak at 29.4°, related to CaCO_3_ (JCPDS 47-1743) likely due to the slight carbonation of calcium ions by CO_2_ present in the atmosphere. The main difference with respect to pure MH concerns the change in the relative intensity among the two main peaks relative to (001) and (101) plane. The intensity of Mg(OH)_2_ (001) plane’s peak, which corresponds to the basal plane of brucite, becomes stronger than the diffraction peak for the (101) plane. As reported in Table 3 Entries 1–7, the intensity ratio of reflections *I*_001_/*I*_101_ increases from 0.78 (MH sample) up to values ranging between 0.95–1.34 for MH-Ca e MH-Ni. No clear correlation is observed between the increase of *I*_001_/*I*_101_ and the metal content. From these results it is possible to conclude that in presence of calcium and nickel, ions preferential growth along the (001) hexagonal basal plane of brucite occurs leading to a layered structure, e.g., flakes or platelets, with high aspect ratio along the *c*-axis. [26,27,28]. Wu et al. have already reported that the strength of (001) plane became stronger than that of (101) plane upon hydrothermal modification of Mg(OH)_2_ in presence of CaCl_2_ [29]. MH-Co samples, instead, shows a peculiar feature. The spectra shown in Figure 3d present reflection peaks, centered at the same position of those of brucite (Figure 3a), with a progressive intensity decrease and peak broadening, as Co content increases, that indicate the lowering of crystallization degree. Rietveld refinement reported in Table 3 confirms that metal ions are included into Mg(OH)_2_ host matrix because a volume cell (*V*(Å^3^)) increase is observed, in relation to the metal load. At lower metal load for all Me-doped samples the volume cell remains almost similar to the MH sample’s one, but increasing Me load it increases till 41.5 Å^3^ for MH-Co3 sample (Entry 9 in Table 3), which presents the higher amount of Co in the matrix. Only for MH-Ni2 and MH-Ni3 (Entries 6 and 7 in Table 3) it decreases. Substituting Mg ion (*r_Mg_*_2+_ = 0.72 Å) with Ca ion (*r_Ca_*_2+_ = 100 Å) it is simple to understand, according to Vegard’s law [30], the cell volume change, while it is more difficult in the case of Co (*r_Co_*_2+_ = 0.70 Å) and Ni (*r_Ni_*_2+_ = 0.70 Å) ions, which ion radius are similar to Mg’s one. In these cases, probably, atoms are substituted interstitially leading to a lattice expansion [31].

**Figure 4:**
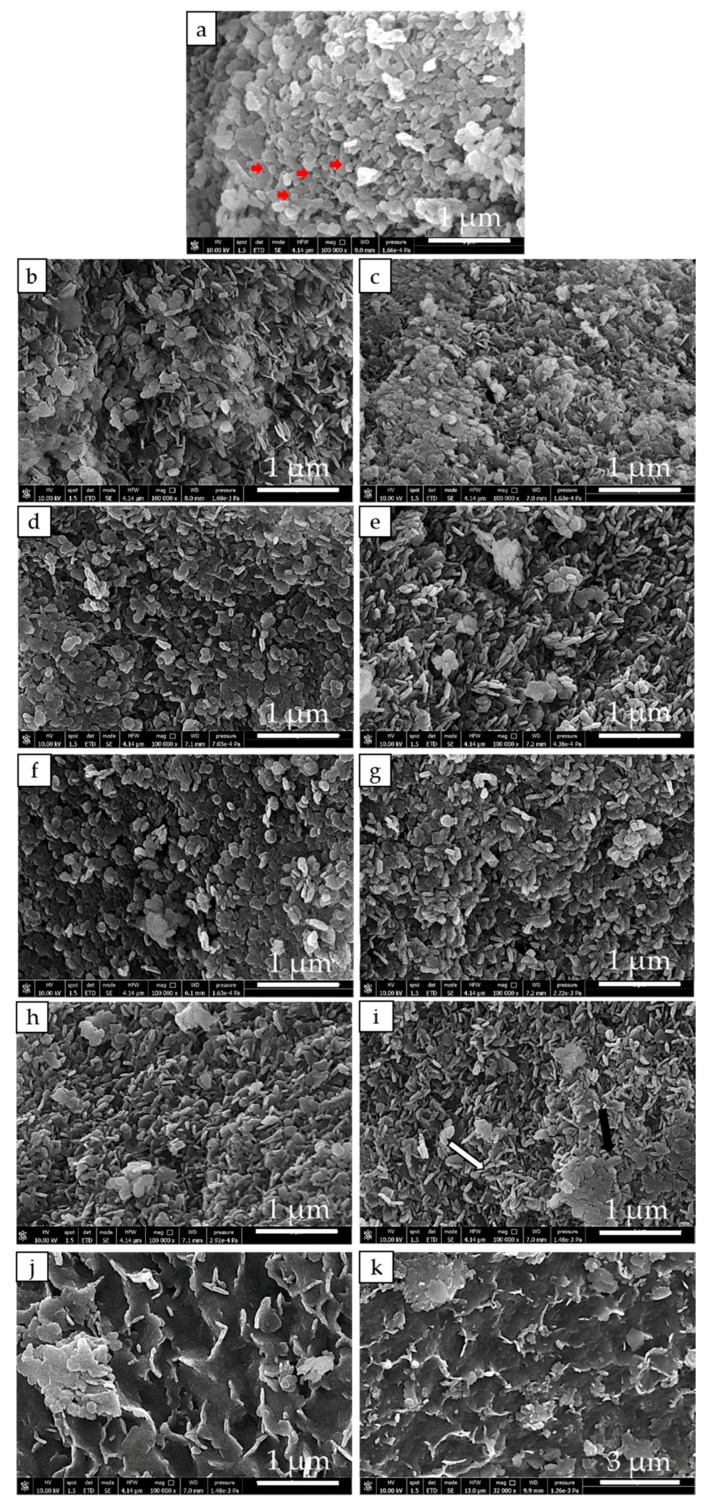
SEM images of investigated samples. MH (**a**); MH-Ca1 (**b**); MH-Ca2 (**c**); MH-Ca3 (**d**); MH-Ni1 (**e**); MH-Ni2 (**f**); MH-Ni3 (**g**); MH-Co1 (**h**); MH-Co2 (**i**); MH-Co3 (**j**,**k**).

The morphology of MH and MH-Me samples is evaluated by means of SEM analysis, shown in Figure 4a–k. MH sample (Figure 4a) presents as large aggregates prevalently formed by magnesium hydroxide hexagonal platelets, in agreement with XRD findings; in addition, a few rounded shaped particles (red arrows) are also visible. The evolution of the sample morphology as the result of the doping by calcium and nickel appears very similar. In particular, increasing the amount of calcium and nickel in the solid, large agglomerates of highly stacked hexagonal brucite particles form (Figure 4b–g). The use of cobalt as doping ion, instead, gives rise to a dramatic change in the morphology with respect to MH, especially at higher cobalt load. Indeed, while for MH-Co1 brucite platelets having the peculiar stacked configuration are still visible (Figure 4h), in case of MH-Co2 and MH-Co3 it is clearly observed the progressive formation of amorphous hydroxide. In particular, for MH-Co2 sample it can be seen magnesium hydroxide platelets (Figure 4i white arrow) embedded into large portions of badly crystallized material (Figure 4i black arrow). Increasing the cobalt content, MH-Co3 sample, the crystalline hexagonal brucite is practically not visible anymore or it is very difficult to distinguish, and large sheets of poorly crystallized hydroxide represent the material’s main component (Figure 4j). SEM analysis is in agreement with the XRD results that evidence the progressive amorphization of Mg(OH)_2_ increasing the cobalt content (Figure 4h–j). 

It is noteworthy from the low magnification images of MH-Co3 sample (Figure 4k) that the large sheets of unshaped badly crystallized hydroxide are very densely packed, forming a continuous and extended rough surface.

**Figure 5:**
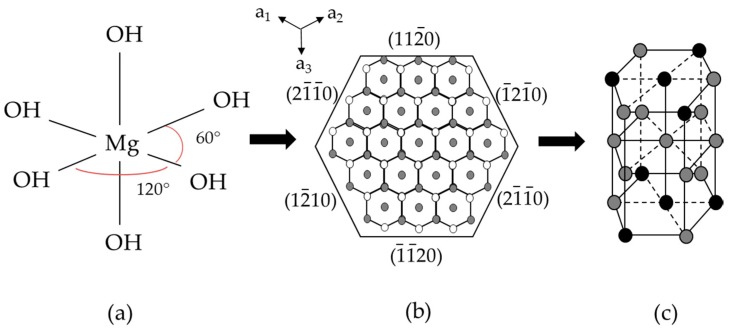
Mg(OH)_2_ growth, based on the model of anion coordination polyhedron (ACP). Growth unit (**a**); Large dimension growth units in the same face (**b**); Hexagonal structure (**c**).

Referring to the mechanism of hexagonal Mg(OH)_2_ growth, based on the model of anion coordination polyhedron (ACP) [32] where the nucleation seeds Mg(OH)_6_^4^^−^ first form the growth units (Figure 5a) that pile up with each other forming large dimension growth units in the same face (*x*, *y*) (Figure 5b) which then connect one to another along the *z* axis forming (001) planes (Figure 5c) and finally the hexagonal structure. It can be argued that calcium and nickel ions promote the growth along the *z* axis (Figure 5c) then the hexagonal structure, as inferred by the increase of the intensity ratio *I*_001_/*I*_101_ while cobalt ions, instead, strongly hinder the piling of growth units in the *x*, *y* plane and then the crystal formation.

The mean particle size of investigated samples, as inferred by DLS analysis, are reported in Table 3.

MH shows the highest value centered at 181 nm. A decrease of the mean particle size is obtained for all MH-Me1 (Entries 2, 5, 8). At higher Me content, two different behaviors have been observed depending on the type of metal. For calcium and nickel doped samples mean particle size returns progressively to increase, although it is lower than that of MH, with the metal content (Entries 3, 4, 6, 7). For MH-Co2 instead, mean particle size continues to decrease in MH-Co2 (Entry 9) while abruptly increases for MH-Co3 sample, containing the highest metal content (Entry 10). 

It is noteworthy that for MH-Ca and MH-Ni samples DLS data really reflects the size of the crystalline hexagonal platelets which seems to be influenced by the cations content. Wu et al. have already reported an increase of Mg(OH)_2_ particle size increasing the calcium content during hydrothermal treatment of hydroxide [29]. The authors suggest that calcium promotes the formation of Mg(OH)^+^ which may be favorable for the formation of nucleation seeds Mg(OH)_6_^4^^−^ which represents the growth unit for Mg(OH)_2_ growth [32]. A similar effect can be depicted for nickel ion.

In the case of MH-Co2 and MH-Co3 samples instead, DLS analysis likely reflects the size of crystalline hexagonal platelets (very few, especially in the case of MH-Co3) in addition to the size of the amorphous portions of the sample. Considering that the preparation of the samples for DLS analysis provides that they are sonicated, it is likely that, due to the lowest mechanical resistance, amorphous phase is fragmented in an uncontrolled way. Therefore, the resulting particle size observed is not the direct evidence of a such influence of cobalt ion during the Mg(OH)_2_ growth.

**Figure 6:**
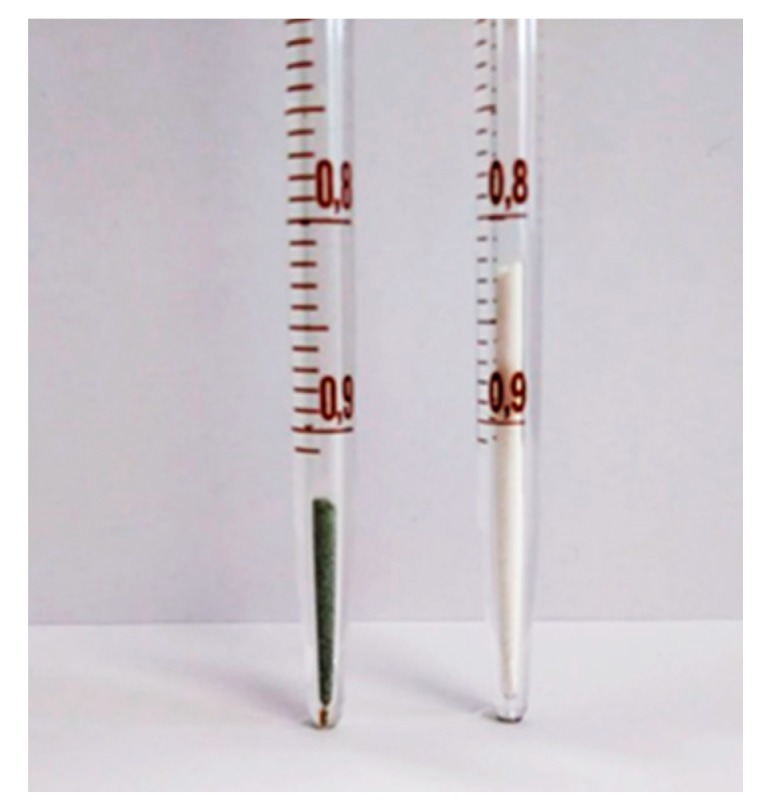
Volume occupied by ~69 mg of MH-Co3 (**on the left**) and MH (**on the right**) samples.

Table 3 also lists the material’s properties such as apparent density *ρ* (kg/m^3^). From the reported data it is evident that the apparent density of all MH-Me samples is significantly higher than that of pure MH. In general, the apparent density enhancement ranges between 68% (sample MH-Co2) up to 200% (sample MH-Co3). The strong enhancement of density is visually demonstrated in Figure 6.

Apparent density is defined as the average density of the material and includes the volume of pores within the particle boundary [33]. Generally, the higher the density, the smaller the pore volume in the sample. The almost general behavior of doped samples (MH-Ca and MH-Ni), in fact, reflects a higher density of the material and a lower value of the porosity, except for the MH-Ni1 sample, which morphology (Figure 4e) appears to be less stacked than the samples with the highest metal load and more similar to MH-Co1 and MH-Co2 (Figure 4h,i), which show a comparable pore volume (Entries 8 and 9 in Table 3). The same peculiar morphology was found for Mg(OH)_2_ prepared in the presence of CTAB, which promotes the formation of well separated Mg(OH)_2_ particles, lowering the hydroxide mean particle diameter and increasing the bulk density likely due to the peculiar stacked configuration of hydroxide particles, reported elsewhere [22].

The increase of apparent density could be due to two concomitant effects, which are the lowering of particle size and the strong agglomeration of magnesium hydroxide particles (MH-Ca and MH-Ni sample) or, as in case of MH-Co, to the densely packed amorphous material formation, as evidenced by SEM and by the lowest value of pore volume (0.245 cm^3^/g) detected for MH-Co3 sample. 

### 3.3. Thermochemical Behavior

**Figure 7:**
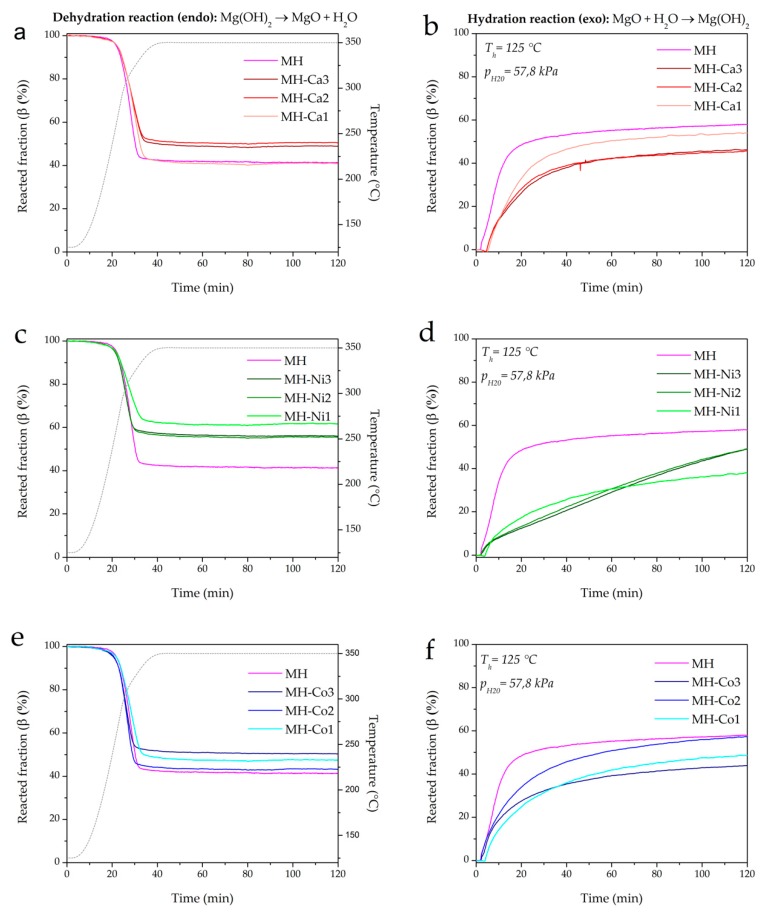
TG analysis, influence of metal loading. Reacted fraction in dehydration and hydration reactions of MH-Ca (**a**,**b**), MH-Ni (**c**,**d**), MH-Co (**e**,**f**).

The thermogravimetric data are calculated assuming the metal doping negligible and Mg(OH)_2_ at 100 wt.%. The curves in Figure 7 are relative to the third cycle, when the thermochemical behavior of the samples was observed to be stable [24].

**Figure 8:**
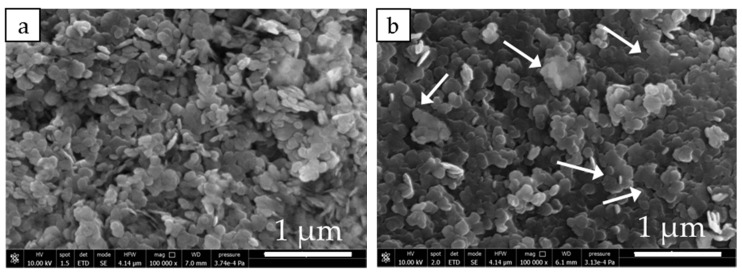

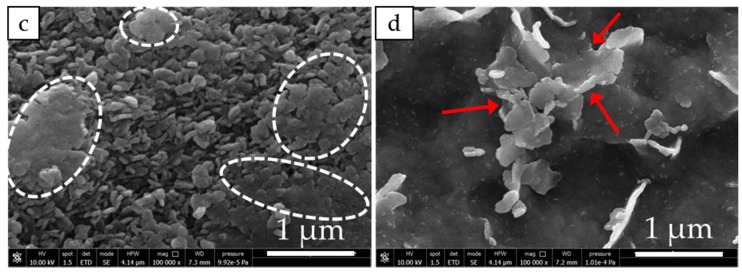
SEM images of investigated samples after cycling: MH (**a**); MH-Ca3 (**b**); MH-Ni3 (**c**); MH-Co3 (**d**).

For all the doped materials, the percentage of MH reacted fraction during dehydration and hydration decreases (Figure 7). MH-Ca and MH-Ni follow the opposite trend observable for morphological properties in Table 3. In fact, Mg(OH)_2_ conversion progressively decreases, increasing metal load from MH-Ca1 to MH-Ca2 (Entries 2 and 3 in Table 4), and then it remains almost stable for MH-Ca3 (Entry 4 in Table 4). The same behavior is observed for MH-Ni. The mean particle size, as described before, progressively increases following the same criteria. For MH-Co1, instead, conversion (%) continues to increase to MH-Co2 (Entry 9 in Table 4) while abruptly decreases for MH-Co3 sample, which presents the highest mean particle size (Entry 10 in Table 3). Additionally, for hydration, a similar trend is observed. If the 1st cycle dehydration reaction is analyzed, it can be observed that the conversion percentage of all the samples is equal to MH conversion and in some cases also higher (Entries 2, 3, 5, 6 and 7 in Table 4) or quite low (Entries 4 and 10 in Table 4). Therefore, the limiting process that influences the material behavior is the hydration. The *β_h_*%, during first cycle, as shown in Table 4, does not reach MH hydration with the exception of MH-Ca1 (Entry 2 in Table 4), which also maintains the higher conversion percentage in the following cycles. This behavior seems to be related to the main particle size reported in Table 3. As discussed above, the smaller particle size is strictly correlated with the higher density of the doped samples. This morphology strongly influences the magnesia hydration. As reported by Tang et al. [34], MgO hydration process follows common MgO dissolution/Mg(OH)_2_ precipitation mechanism, well accepted in the literature [35,36,37]. Initially water vapor is chemisorbed on the MgO and then physically adsorbed to form a liquid layer on the surface of the solid (chemical control of the reaction). This layer of water reacts with the MgO to form a surface layer of Mg(OH)_2_, that covers surfaces and pores of MgO particles. As a result, the diffusion of water vapor is hindered inside the particles, which reduces the overall reaction rate and the rehydration conversion *β_h_*% (diffusion controlled). When density is high, because of the small particle size and the packed morphology described in Figure 4, the porosity of the material is very poor and the water permeability is difficult. Figure 8 shows the SEM analysis of the investigated samples after cycling. For brevity, only MH and doped samples with highest metal load are reported (MH-Ca3, MH-Ni3, MH-Co3). It can be observed that coalescence is more favored for doped samples. For MH-Ca3 and MH-Ni3, the particle size increase is clearly observable (compare Figure 4 (white arrows) with Figure 8 (white circles)), MH-Co3 keeps its packed structure, formed by large sheets of poorly crystallized hydroxide (red arrows in Figure 8). Probably, also in this case, the high density plays a very important role influencing the change of morphology during the dehydration/hydration cycles. The presence of a lower porosity of the material and a smaller particle size favors the coalescence of the latter in larger particles; this decreases heat transfer property and leads to further loss of bulk porosity diminishing the MgO rehydration kinetics [24].

Also noteworthy is the fact that, from the slope of the dehydration and hydration curves, doped samples exhibit similar dehydration kinetics with respect to un-doped MH (see Figure 7a,c,e). On the contrary, hydration kinetics is highly affected by the doping, which, in general, decreases the kinetics (see Figure 7b,d,f). This is evident especially in Ni-doped samples (see Figure 7d). Hence, depending on the final application of the storage technology, heat can be released at a required rate by tuning MH with a proper dopant cation and amount. 

**Figure 9:**
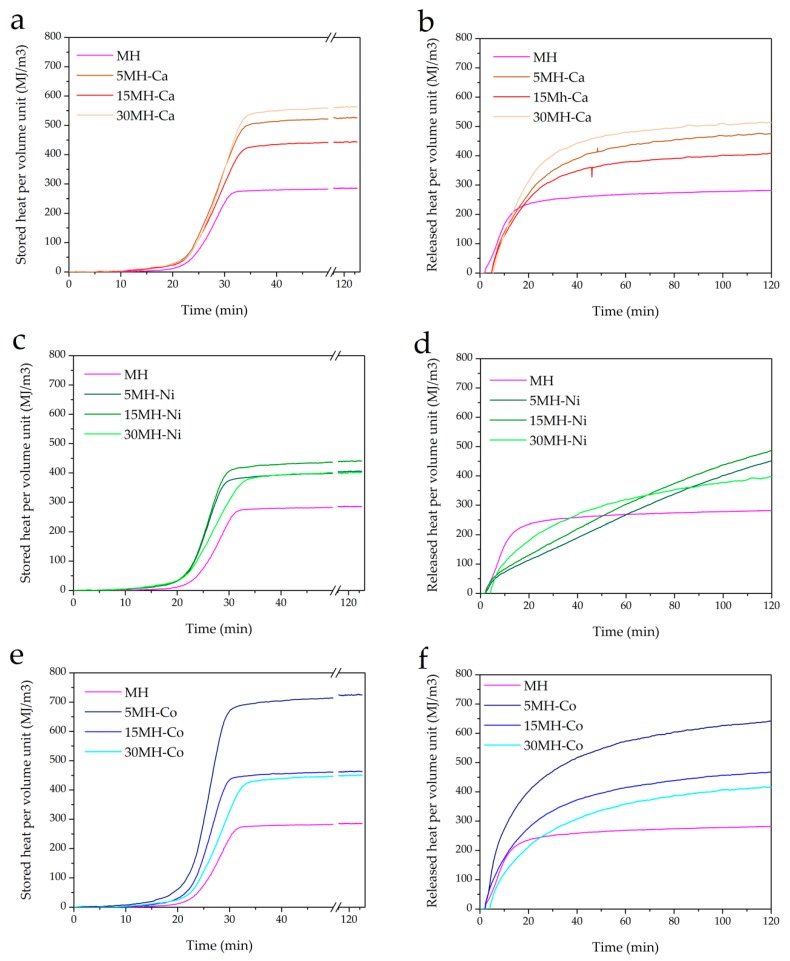
Stored and released heat per volume unit of MH-Ca (**a**,**b**), MH-Ni (**c**,**d**), MH-Co (**e**,**f**).

Looking at stored/released heat capacity by unit volume (*Q*_*s*/*r*_*^V^*) (Figure 9a–f) it can be seen that MH shows the lowest stored/released heat capacity that is 285 MJ/m^3^ with respect to MH-M samples for which a higher *Q*_*s*/*r*_*^V^* is generally observed, as a consequence of the higher apparent density (Table 3). 

The highest value 725 MJ/m^3^ is achieved on MH-Co3 (Figure 9e), which is almost three times higher than MH’s value. This value is, so far, the highest reported in the literature for pure Mg(OH)_2_ heat storage material. The stored heat increases with an increase in the metal load doping. Released heat per volume unit in doped samples almost never reaches the 100% of stored heat.

## 4. Conclusions

The present study clearly suggests that morphological characteristics (porosity, mean particle size) and apparent density are significantly influenced by the Me (Ca^2+^, Ni^2+^, Co^2+^) doping during the preparation of Mg(OH)_2_ through precipitation. It was found that, considering that no calcium, cobalt and nickel hydroxides precipitate during the synthesis, these metal ions are included into Mg(OH)_2_ host matrix, as confirmed by Rietveld refinement of XRD analysis. All the investigated samples show an apparent density increase. MH-Co3, which presents badly crystallized and highly packed hydroxide, reaches a higher density than the MH sample of 200%. Apparent density describes two concomitant effects that are the lowering of particle size and the strong agglomeration of magnesium hydroxide particles (MH-Ca and MH-Ni samples) with a consequent decrease in sample porosity. In MH-Co case, the high density is due to a densely packed amorphous material formation. A correlation between morphological properties and the thermochemical behavior of Mg(OH)_2_ is found. In particular, for all the investigated doped samples a lower reacted fraction is obtained in comparison with the not-doped material. However, because of the higher apparent density, the doped samples exhibit higher volumetric stored/released heat capacity. The highest value is reported for MH-Co3 sample (725 MJ/m^3^), and it is almost three times higher than MH’s value. In future development, the doped samples will be further investigated to enhance their performance, while maintaining the high density, and they will be tested for several cycles, to investigate their stability in real applications. 

## Figures and Tables

**Table 1:** Sample code, chemical compositions of the solutions. Mg^2+^ and OH^−^ molar concentration were 0.01 M and 0.063 M in each preparation.

Sample Code	Type of Me^2+^	[Me]	Me/Mg^2+^ Nominal Molar Ratio
MH	-	-	-
MH-Ca1	Ca^2+^	0.0003	0.033
MH-Ca2	Ca^2+^	0.0007	0.067
MH-Ca3	Ca^2+^	0.0020	0.200
MH-Ni1	Ni^2+^	0.0003	0.033
MH-Ni2	Ni^2+^	0.0007	0.067
MH-Ni3	Ni^2+^	0.0020	0.200
MH-Co1	Co^2+^	0.0003	0.033
MH-Co2	Co^2+^	0.0007	0.067
MH-Co3	Co^2+^	0.0020	0.200

**Table 2:** Evaluation of supersaturation conditions for Mg(OH)_2_, Co(OH)_2_, Ni(OH)_2_ and Ca(OH)_2_ formation under conditions used in the present work. Y: Yes, N: No.

Hydroxide	[Me^2+^] [OH^−^]*^n^*	K_sp_ (at 25 °C)	Supersaturation Condition
Mg(OH)_2_	3.98 × 10^−^^7^	1.80 × 10^−11^	Y
Ca(OH)_2_	1.33 × 10^−^^8^	7.90 × 10^−06^	N
Ca(OH)_2_	2.65 × 10^−^^8^	7.90 × 10^−06^	N
Ca(OH)_2_	7.96 × 10^−^^8^	7.90 × 10^−06^	N
Ni(OH)_2_	1.33 × 10^−^^8^	2.80 × 10^−16^	Y
Ni(OH)_2_	2.65 × 10^−^^8^	2.80 × 10^−16^	Y
Ni(OH)_2_	7.96 × 10^−^^8^	2.80 × 10^−16^	Y
Co(OH)_2_	1.33 × 10^−^^8^	2.50 × 10^−16^	Y
Co(OH)_2_	2.65 × 10^−^^8^	2.50 × 10^−16^	Y
Co(OH)_2_	7.96 × 10^−^^8^	2.50 × 10^−16^	Y

**Table 3:** Intensity ratios and morphological properties of investigated samples.

Entry	Sample Code	Intensity Ratios		Rietveld Refinement	Morphological Properties		
		*I*_001/101_	*I*_001/110_	*V*(Å^3^)	Mean Particle Size (nm) *	*ρ* (kg/m^3^)	*V_pore_* (cm^3^/g)
1	MH	0.78	2.63	40.9	180.5 ± 24.0	350	0.618
2	MH-Ca1	1.16	3.64	40.7	78.9 ± 44.5	685	0.614
3	MH-Ca2	1.34	4.77	40.8	131.3 ± 33.1	644	0.497
4	MH-Ca3	1.04	3.57	41.3	117.1 ± 45.6	740	0.525
5	MH-Ni1	1.09	3.26	40.8	97.8 ± 50.1	752	0.885
6	MH-Ni2	0.97	3.19	41.3	105.8 ± 38.1	712	0.478
7	MH-Ni3	0.95	3.26	41.0	118.4 ± 20.6	663	0.515
8	MH-Co1	0.89	2.36	40.4	85.8 ± 34.0	616	0.778
9	MH-Co2	0.72	1.69	40.9	67.1 ± 40.0	587	0.914
10	MH-Co3	1.85	4.84	41.5	188.6 ± 23.4	1.050	0.245

* Measured by means of Dynamic Light Scattering analysis.

**Table 4:** Comparison between dehydration/hydration conversions (Δ*β*_*d*/*h*_) at first and third cycles.

Entry	Sample Code	1st Cycle		3rd Cyle		Q_s_^V^ (MJ/m^3^)	Q_s_^V^ (MJ/m^3^)
		*β_d_* (%)	*β_h_* (%)	*β_d_* (%)	*β_h_* (%)		
1	MH	89.0	61.6	58.8	58.0	285.4	281.95
2	MH-Ca1	90.0	62.7	58.8	54.0	536.4	513.98
3	MH-Ca2	90.0	52.7	49.4	45.6	444.9	408.44
4	MH-Ca3	88.3	55.8	51.2	45.6	525.7	475.5
5	MH-Ni1	93.7	36.6	38.2	38.0	401.7	397.01
6	MH-Ni2	97.7	42.0	44.4	49.0	441.0	485.01
7	MH-Ni3	94.8	41.1	44.4	49.0	407.0	449.36
8	MH-Co1	89.0	55.5	52.5	48.6	450.6	416.22
9	MH-Co2	89.0	56.6	56.8	57.2	463.3	466.98
10	MH-Co3	87.2	55.5	49.7	43.8	724.8	640.75
